# Thermal Pulse Processing of Blanks of Small-Sized Parts Made of Beryllium Bronze and 29 NK Alloy

**DOI:** 10.3390/ma15196682

**Published:** 2022-09-26

**Authors:** Madina E. Isametova, Nikita V. Martyushev, Yuliya I. Karlina, Roman V. Kononenko, Vadim Y. Skeeba, Bakhyt N. Absadykov

**Affiliations:** 1 Department of Mechanical Engineering, Institute of Energy and Mechanical Engineering, Satbayev University, KZ-050000 Almaty, Kazakhstan; 2 Department of Advanced Technologies, Tomsk Polytechnic University, 634050 Tomsk, Russia; 3 A. P. Vinogradov Institute of Geochemistry of the Siberian Branch of the Russian Academy of Sciences, 664033 Irkutsk, Russia; 4 Computer Hardware and Software Laboratory, Institute of Information Technologies and Data Analysis, Irkutsk National Research Technical University, 664074 Irkutsk, Russia; 5 Department of Industrial Machinery Design, Novosibirsk State Technical University, 20, K. Marks Ave., 630073 Novosibirsk, Russia

**Keywords:** thermal pulse machining, beryllium bronze machining, 29 NK alloy machining, deburring

## Abstract

The presence of burrs on parts is not allowed in high-tech industries; there is a tendency to improve accuracy and quality and to reduce overall dimensions. A high proportion of operations are aimed at removing burrs in the labor intensity of release. Thermal pulse deburring machines are being developed and are applicable for deburring small-sized high-precision parts while providing additional processing conditions. A significant part of the electronic component base—coaxial radio components—is produced from beryllium bronze and the 29 NK alloy. It is not possible to prevent burr formation when cutting these materials. The conditions for deburring by the thermal pulse method are established in compliance with the requirements for deviations in the geometry of parts, for surface roughness and for ensuring maximum processing performance. These are restrictions on the thickness of the burr root, a variant of the arrangement of parts in the chamber of thermal impulse installation, which ensures the prevention of damage to parts during processing. Additionally, it provides access to a combustible mixture of all the surfaces of the parts; there is also a pressure value of the combustible mixture, depending on the characteristics of the thermal pulse installation, the total area of the treated surface, and the thermal conductivity of the materials for workpieces.

## 1. Introduction

In many high-tech industries, such as aerospace, instrument-making, and military-industrial complex, the presence of burrs on parts is not allowed [1,2]. The tendency to improve accuracy and quality and to reduce the overall dimensions of products necessitate minimizing the size of burrs and improving technologies for their removal. Thus, the share of deburring operations in the radio-electronic industry reaches up to 50% of the labor intensity of part production. Refusal to use manual locksmith operations to remove burrs under a microscope significantly reduces the labor intensity of the production of parts and increases labor productivity.

The intensification of the process for obtaining high-quality small-sized high-precision parts, typical of the enterprises of the radio-electronic industry, is based on a new approach of two-stage deburring with an increase in productivity and quality of part processing. Two-stage deburring is a cutting process used to obtain the minimum size of the burr root, and subsequent deburring is carried out by the advanced thermal pulse method.

The patents for thermal pulse installations and studies on thermal pulse deburring describe systems for high-precision dosing and control of processing modes, which makes it possible to use the method for high-precision parts. Much research has been devoted to deburring large- and medium-sized parts. The use of the thermal pulse deburring of small-sized high-precision parts has its own characteristics. The condition for successful removal of burrs from such parts by the thermal pulse method is to ensure acceptable burr sizes at the cutting stage.

A set of studies has been carried out by the example of producing parts for coaxial radio components from beryllium bronze and the 29 NK alloy on CNC machines. Coaxial radio components are part of the electronic component base for microwave microelectronics, and they consist of inner and outer conductors, as well as an insulator between the conductors [1]. For sealed connectors, the cases and inner conductors are made of the 29 NK alloy to obtain thermal expansion coefficients of junctions with the S52-1 glass that are consistent in magnitude. For leaky connectors, the conductors are made of beryllium bronze, which has elasticity, allowing one to tightly compress the socket lamellas [1].

According to the industry standard for parts of electronic equipment, all workpieces must be cleaned of scale, burrs must be removed, and sharp edges are to be blunted. Insufficient preparation of the surface of the parts before coating (presence of burrs, sharp edges, contamination) leads to adhesion deterioration and the formation of outgrowths on the surface of the parts [2,3]. To preserve the electrical conductivity of products, it is necessary to ensure full contact of the mating surfaces during assembly. Burrs and dirt lead to surface damage during assembly, which results in a decrease in the operational characteristics of electronic equipment. The sharp edges of parts become stress concentrators, entailing the destruction of their surfaces [1,2,3]. The problem of burr formation in the cutting step makes it necessary to remove the burr. The applied deburring methods may cost differently depending on the amount and nature of the burrs. The instability of cutting technology in terms of burr generation leads to uncertainty in the time and cost of further processing. In this regard, the problem of producing small-sized parts made of beryllium bronze and epy 29 NK alloy without burrs with minimal costs is an urgent task. Despite the existing groundwork, the problem of deburring has not been studied sufficiently.

On the basis of a literature review, it has been established that it is not possible to completely prevent the appearance of burrs at the stage of cutting materials, such as beryllium bronze and the 29 NK alloy. The level of development of the thermal pulse deburring method, gas mixture dosing control systems, and pressure and temperature control allows it to be used for deburring small-sized high-precision parts. The works of the following scientists are devoted to the study of the processes of thermal impulse processing: L.K. Gillespie, N.I. Pak, S.A. Shikunov, S.I. Adoniy, V.I. Manzhalei, A.V. Losev, S.I. Plankovsky, O.V. Shipul, O.V. Trifonov, O.S. Borisova, J.K. Paik, and I.S. Manuilovich. To study the processes occurring in parts during thermal pulse processing, classifiers of defects and edge distortions have been developed. Gillespie [4] divided burrs into four types: Poisson burrs, curling burrs, torn burrs, and cut burrs. When cutting, cutting and hanging burrs are formed. The following burr parameters are determined: dimensions, hardness, configuration along the edge, cross-sectional shape, and location. The parameters of thermal pulse processing are influenced by such a factor as the root thickness. A number of works were carried out at the Krasnoyarsk Branch of the Siberian Branch of the Russian Academy of Sciences. In the work [5], N.I. Pak and S.A. Shikunov analyzed the possibility of removing burrs from the surface by evaporation and reflow, and they concluded that burrs are impossible to remove by evaporation without carrying away the main material of the part. In the works [6,7], S.I. Adonin and V.I. Manzhalei carried out experimental and theoretical studies on heating models of burrs in the form of a wedge, plate, and wire by the radiation of detonating gases during melting and combustion. The attenuation regularity of shock waves in combustion chambers of the constant volume with a height-to-diameter (H/D) ratio being less than or equal to 5 has been established by Losev in [8,9,10]. When processing the workpieces made of the 29 NK alloy, containing 29% of nickel, it must be taken into account that its thermal conductivity increases with increasing temperature. Modeling was carried out on the basis of regression analysis or the method of selecting functions that best suit the experimental data. Models have not been created for all mechanisms of burr removal (melting, combustion in excess of an oxidizing agent, brittle shearing) depending on the processing modes (power of the heat source, its duration), geometric parameters of the burr, and material. The numerical modeling of the nature of pressure changes during the combustion of a gas-air mixture in a closed chamber carried out on the basis of an experimental study in the LRET laboratory of Busan National University (South Korea) is described in [11]. S.I. Plankovsky, O.V. Shipul, O.V. Trifonov, and O.S. Borisova conducted similar studies, taking into account the heterogeneity of the fuel mixture, at the Kharkov Aviation Institute [12]. The simulation was carried out without taking into account the transition of combustion to the detonation regime. Explosive, detonation processes in channels and open space are studied by I.S. Manuilovich in [13]; the study was carried out using the experimental setup of the Research Institute of Mechanics, Moscow State University. Original methods of initiating detonation associated with the movement of solid surfaces limiting the flow area and data on the influence of the shape of the cylindrical combustion chamber of a pulsating detonation engine on its traction characteristics are obtained. S.I. Plankovsky, O.V. Shipul, O.V. Trifonov, and V.G. Kozlov [14] developed a mathematical model for the transition of combustion of fuel mixtures, based on methane and propane, to the detonation mode using a simplified one-stage combustion model. Losses due to heat exchange with the chamber body, which have a significant impact when processing parts having a complex surface from materials with high thermal conductivity, are not taken into account. The analysis of the studies showed that the created models of the process of deburring by the thermal pulse method do not take into account all the factors that affect the process and are also quite difficult to use due to the presence of 25 factors, the combination of which affects the calculation result. Studies reflecting the influence of cutting modes on the parameters of burrs and the conditions necessary for the subsequent thermal pulse processing of small-sized high-precision parts have not been considered enough.

The purpose of this study is to increase the efficiency of the deburring process by creating favorable conditions for physical and technical thermal pulse processing of small-sized high-precision parts made of BrB2 beryllium bronze and the 29 NK alloy.

To achieve this goal, the following tasks were solved:

(a) Evaluation of the influence of the turning mode parameters of small-sized high-precision parts made of beryllium bronze BrB2 and the 29 NK alloy on the size of the roots of the burrs formed to ensure the minimum size of the roots.
(b) Experimental establishment of rational parameters of thermal pulse processing of parts, ensuring the removal of burrs.
(c) Development of a rational option for filling the chamber of the thermal impulse installation with parts to ensure high-performance deburring without damaging the surfaces of the parts.

The object of the study is the process of blade and thermal pulse processing of small-sized high-precision parts of coaxial radio components made of beryllium bronze of the BrB2 brand and the 29 NK alloy with a diameter between 0.4 and 10 mm and a length ranging from 4 to 15 mm.

The subject of the study is the relationship between the parameters of the turning mode of workpieces and the parameters of the remaining burrs, as well as the relationship of the size of the burrs with the modes and performance of thermal pulse deburring.

## 2. Materials and Methods

The parts manufactured on CITIZEN CINCOM CNC precision longitudinal turning lathes were from such materials as beryllium bronze Rod BrB2 (GOST 15835-2013) and the precision 29 NK alloy with a given temperature coefficient of linear expansion (Circle 6-B-h7, GOST 149*55-77 29 NK, GOST 14082-78). These materials were difficult to cut due to their physical and chemical properties. To study the nature, causes of burrs, and the methods of their removal, analytical and experimental research methods were used. Experimental methods were applied as full-scale experiments representing an experimental selection of cutting modes and tools in which the thickness of the burr root does not exceed the established norm. There was an experiment on deburring in a PULSAR VKF 3.250 thermal pulse installation, manufactured by AlfaSteel, St. Petersburg, Russia. The characteristics of burrs were studied under a microscope using the provisions of the theory for metal cutting and analysis of factors affecting the degree of burr removal during thermal pulse surface treatment of parts. Burr characteristics and dimensions were analyzed under a SEM Multi Beam System JEOL JIB-Z4500 scanning electron microscope, produced by the company JEOL, Tokyo, Japan. The length and thickness of the largest burr root on the sample was calculated based on the image taken on this SEM with a resolution of 2.5 nm at a 350× magnification. The roughness was measured in a non-contact way using an optical profilometer ContourGT-K, manufactured by Bruker Nano Inc., Billerica, MA, USA. The nominal values of the roughness parameter Ra varied from 0.005 to 100 µm. The limits of the permissible standard deviation of the parameter were Ra ± 3%. The thread dimensional parameters were controlled using the through and non-through threaded ring gage.

## 3. Results

### 3.1. The Influence of the Parameters of the Turning Mode of Small-Sized High-Precision Parts, Made of Beryllium Bronze BrB2 and the 29 NK Alloy, on the Size of Burr Roots

By the example of the body details of the coaxial radio component made of beryllium bronze and the bushing made of the 29 NK alloy, which have a complex geometry, cutting tools for turning, drilling, and processing modes were selected, in which the processing results meet the criteria of rationality. The selected values of processing modes and the ranges of values recommended by the tool manufacturer were compared. The best results were obtained when using precision cutting tools for micromechanics of the Swiss companies Utilis (Muellheim, Germany), Fraisa (Bellach, Switzerland), IFANGER (Uster, Switzerland), and Applitec (Moutier, Switzerland). This tool has high wear resistance and high thermal conductivity required to reduce the temperature in the cutting area. Its geometry provides a combination of the shape of the surfaces of the cutting part and angle values, which contributes to the sufficient strength of the cutting wedge of the tool, required quality of the machined surface, and minimum cutting forces. To reduce the temperature in the cutting area, processing is carried out using an oil cutting fluid.

The process of burr formation on the parts of coaxial radio components during cutting is affected by the physical and mechanical properties of such materials as beryllium bronze and the 29 NK alloy. These are Young’s modulus, strength, and thermal conductivity, as well as technological parameters, such as the degree of blade wear, processing mode parameters (cutting depth, longitudinal feed, cutting speed), tool geometry, use of a cutting fluid, and rigidity of the machine-tool-tool-part system.

**Figure 1:**
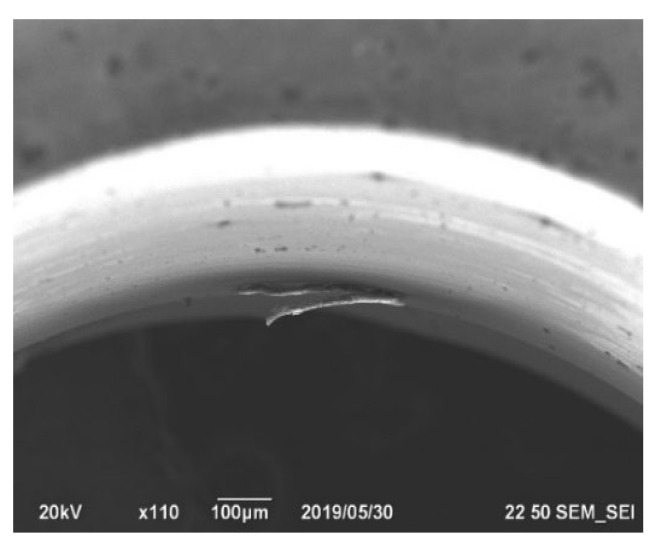
The nature of the burrs on the details of beryllium bronze with an increase in the cutting speed and feed: There is a strong attachment to the surface of the part along the length of the burr.

The main parameters are described, and the nature of the burrs is investigated. The materials used for the manufacture of parts of coaxial radio components are subject to processing by the thermal pulse method. Beryllium bronze and the 29 NK alloy were oxidized. They have different thermal conductivity. The thermal conductivity of beryllium bronze is λ = 75.3 W/(m K) [15], and the thermal conductivity of the 29 NK alloy is λ = 17 W/(m K) [16]. During turning and drilling, burr formation occurs in the feed direction of the cutting edge of the tool. The cutting force deforms the workpiece material. The main factors in the process of burr formation are conditioned by the properties of the materials. Due to the high ability of beryllium bronze to resist stretching and compression under elastic deformation during turning, a dense drain chip occurs, which contributes to the formation of burrs. The process of burr formation during turning of workpieces from the 29 NK alloy is mainly affected by its hardness. To reduce the influence of the thermal conductivity of these materials on the formation of burrs during turning, an oil cutting fluid is used. According to the equipment manufacturer, the root thickness of the removed burrs from the parts made of brass and beryllium bronze should not exceed 0.1 mm, and it must not exceed 0.3 mm on the parts made of the 29 NK alloy. Taking into account the thinness of the wall, the thickness of the burr root on the parts of coaxial radio components should not exceed 0.088 mm. Then, the parts made of such materials as beryllium bronze, brass, and 29 NK alloy are combined into one group to select the modes of thermal pulse processing. The appearance of long stretching burrs with a large attachment area along the burr to the surface of the part is critical for thermal pulse deburring. The thickness of burr root reaches 0.1 mm (Figure 1).

**Figure 2:**
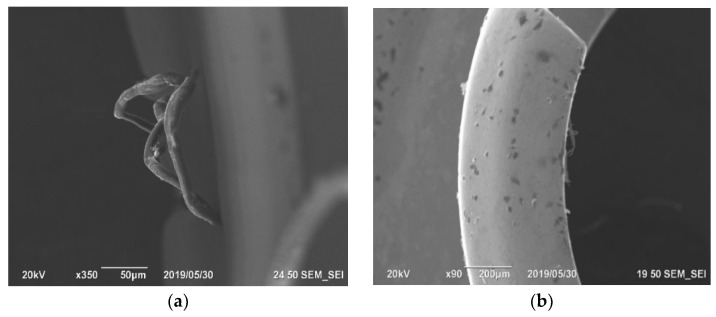
Change in the nature of burrs on parts made of beryllium bronze: (**a**) processing with the parameters recommended by the tool manufacturer (upper values); (**b**) processing the experimentally selected parameters.

When processing the experimentally selected parameters, the burrs are short and drop-shaped; the root thickness is up to 0.03 mm. With an increase in the cutting speed up to 125 m/min and feed up to 0.1 mm/rev, the burrs are long and stretching; the root thickness is up to 0.08 mm (Figure 2). The burrs on beryllium bronze workpieces are highly resilient. The burrs on workpieces made of the 29 NK alloy have a high hardness. The length and thickness of the burr root increase along with tool wear 1.5–1.7 times.

### 3.2. Rational Parameters of Thermal Impulse Processing of Parts, Ensuring the Removal of Burrs without Damaging the Surfaces of Parts

**Figure 3:**
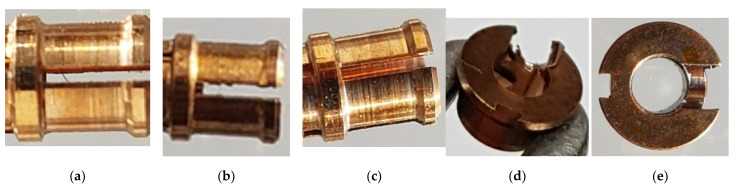
Removal of burrs from the parts made of BrB2 in the Pulsar VKF 3.250 thermal pulse installation: (**a**) the part before processing with burr in the slot of the lamella (the dimensions are D = 3.1 mm, L = 6.5 mm); (**b**) the part after processing, the roots of burrs remained in the slot of the lamella; (**c**) the part after processing in a rational mode, burrs with a root thickness of less than 0.07 mm are removed, and the surface quality and dimensions are preserved; (**d**) the part with burrs with the root thickness less than 0.1 mm (the dimensions are D = 4 mm, L = 2 mm) before processing; (**e**) the part after double processing in a rational mode with burrs removed and matte surface and dimensions saved.

**Figure 4:**
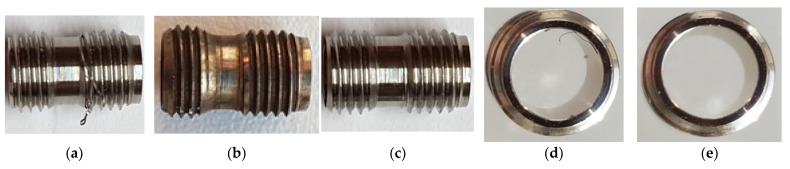
Removal of burrs from the parts made of the 29 NK alloy in the Pulsar VKF 3.250 thermal pulse installation: (**a**,**d**) the part with long burrs (the dimensions are D = 6 mm, L = 10.2 mm) before processing; (**b**) the part with a burr with a root thickness of 0.1 mm after processing at P = 1.8 MPa; the surface is scorched, the edges of the thread are rounded, and the burr is removed; (**c**,**e**) the part after processing in the rational mode with burrs removed and surface quality and dimensions preserved.

An experiment was carried out to remove burrs in a laboratory thermal pulse unit, Pulsar VKF 3.250 produced by Alfa Steel, St. Petersburg, Russia. Easily damaged parts with hard-to-reach surfaces made of beryllium bronze and the 29 NK alloy were selected for processing. To use the setup for quick placement of parts in the device to fill the chamber with parts, the parts are divided into two groups: one with a diameter between 2 and 4 mm and a length between 2 and 5 mm and the other with a diameter between 5 and 10 mm and a length between 6 and 10 mm. The main parameters that affect the processing by the thermal pulse method are the composition of the gas–oxygen mixture, the combustion temperature, and the volume and pressure of the gas–oxygen mixture. The composition of the gas–oxygen mixture is controlled by the stoichiometric ratio of gas mixing [17]. Oxygen performs two tasks. It is necessary to burn the fuel gas because the fuel gas must react with oxygen and thus consume it to generate heat. One shot of propane with two shots of oxygen is burned to form carbon dioxide and water. This means that if the mixing ratio is $C_{3}H_{8}∶O_{2}=1∶2$, then all the fuel gas reacts with oxygen; a little more oxygen is added to oxidize the burrs. In this case, combustion occurs, and the temperature reaches its maximum. With an excess of oxygen introduced into the deburring chamber, the oxygen can perform its second task: burning off the burrs. The more oxygen available, the greater the material removal. If the oxygen content is too high, then no deburring will occur because the burrs cannot be brought up to the ignition temperature [18]. In the installation for thermal deburring of parts, gases are dosed through a gas metering system. The flow meter ensures accurate dosing of the working medium and reproducible deburring results. Initial processing modes are the combustion temperature of T = 3500 °C, the pressure of P = 1800 kPa, the ratio of gas supply under pressure of $P_{C_{3}H_{8}}=3.70\;\mathrm{MPa}$ and $P_{O_{2}}=6.65\;\mathrm{MPa}$, the combustion time of 20–30 milliseconds, and the propane–oxygen mixing ratio of 1:2. These are stoichiometric parameters. When selecting modes, the pressure is reduced to 1760 kPa since, at a pressure of 1800 kPa, the geometry of thin-walled parts is violated. It has been established that during processing in the rational mode, the surface roughness is slightly reduced, the burrs are completely removed, the part geometry is not changed, and the thread geometry is preserved (Figure 3 and Figure 4).

Figure 3 shows the results of the experiment on removing burrs from beryllium bronze parts in different modes. As can be seen in Figure 3a, the part had long thin burrs with a root thickness of 0.51 mm, which is less than 1/6 of the minimum wall thickness of the part. When the processing mode was set with an insufficiently powerful thermal impulse effect (Figure 3b), burr roots remained on the slot of the part lamella. When setting the rational processing mode (Figure 3c), the burrs are removed, and the surface quality and dimensions of the part are preserved.

Figure 3d,e shows the results of the experiment on deburring by double processing in the Pulsar VKF 3.250 thermal pulse installation of parts from non-thin-walled beryllium bronze that have large burrs with a large root thickness. After processing the part in a rational mode, the burrs are removed, the surface quality and dimensions of the part are preserved, and there is a slight darkening of the surface due to the formation of copper oxides on the surface. This defect is easily eliminated by placing the parts in an ultrasonic bath using chemical solutions, such as phosphoric acid.

The microstructure of the surface layer changes at high temperature, but this does not impair the material properties necessary for the manufacture of parts of coaxial radio components. The elasticity of beryllium bronze is preserved, which is required for tight compression of the lamellas of the nest of leaky connectors. Hermetic junctions of the 29 NK alloy with the S52-1 glass were obtained. Both materials have been successfully electroplated with gold–cobalt 1 to 5 microns thick to form a highly conductive “skin layer” for signal transmission in a coaxial line. Orthophosphoric acid cleans the surface of the workpieces from combustion products after thermal pulse treatment and does not affect the above-described properties of materials.

Figure 4 shows the results of the experiment on removing burrs from the 29 NK alloy parts. Before processing, the part had long lingering burrs that arose during threading (Figure 4a), as well as small, long lingering, and drop-shaped burrs on the inner surface (Figure 4d). When setting the processing mode with an excessively powerful thermal impulse effect and a burr root thickness of 0.1 mm (Figure 4b), the burrs are removed, but the surface is scorched, and the sharp edges of the thread are rounded. After processing the part in a rational mode (Figure 4c,e), burrs were removed from the outer and inner surfaces, and the surface quality and dimensions of the part were preserved.

**Figure 5:**
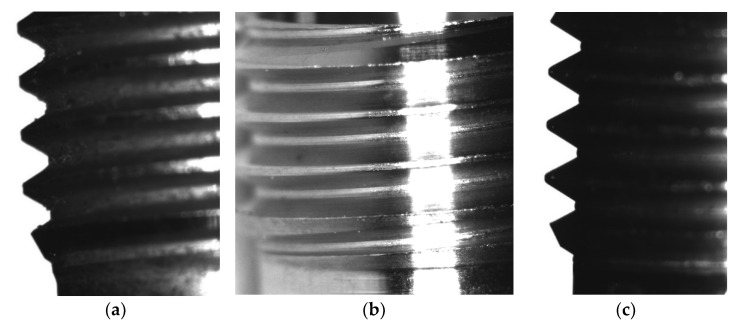
Thermal pulse thread processing: (**a**) the part with burrs before processing; (**b**,**c**) the part after processing in the rational mode of P = 1760 kPa with burrs removed and surface quality and thread dimensions preserved.

Figure 5 shows the result of processing the thread of a part made of the 29 NK alloy, a general view of which is shown in Figure 4. The overall dimensions of the part are the length of 10.2 mm and the diameter of 6 mm.

**Figure 6:**
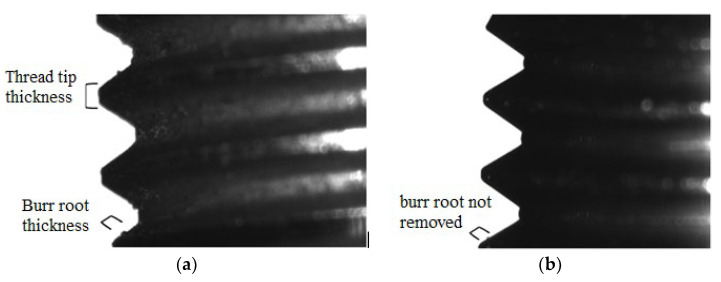
The thickness of the root of the burr is more than 1/4 of the thickness of the thread tip: (**a**) before processing; (**b**) after processing when small burrs are removed; the root of the large one remains, and the top of the thread is preserved.

Figure 6 shows an example of a burr root thickness limitation.

**Figure 7:**
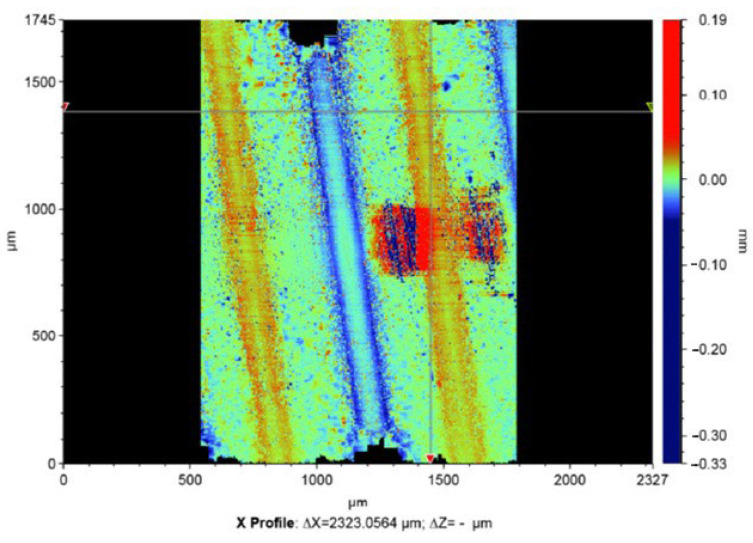
Thread surface quality before thermal pulse processing.

**Figure 8:**
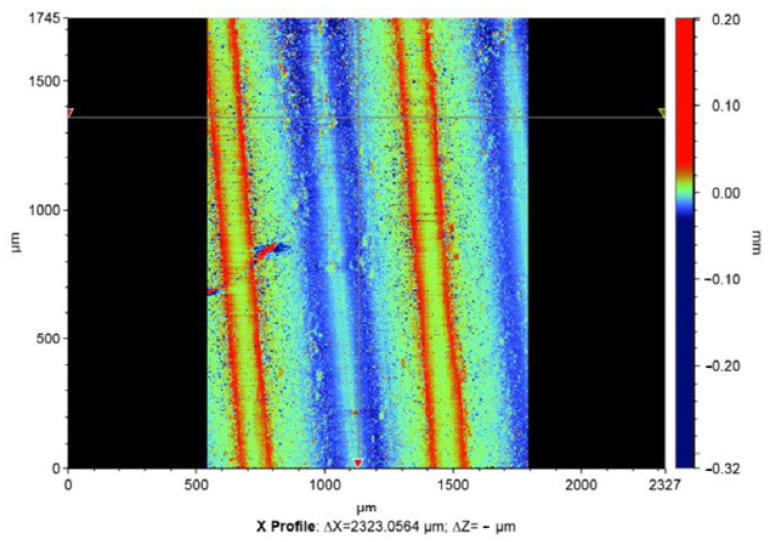
Thread surface quality after thermal pulse processing in the rational mode of P = 1760 kPa.

**Figure 9:**
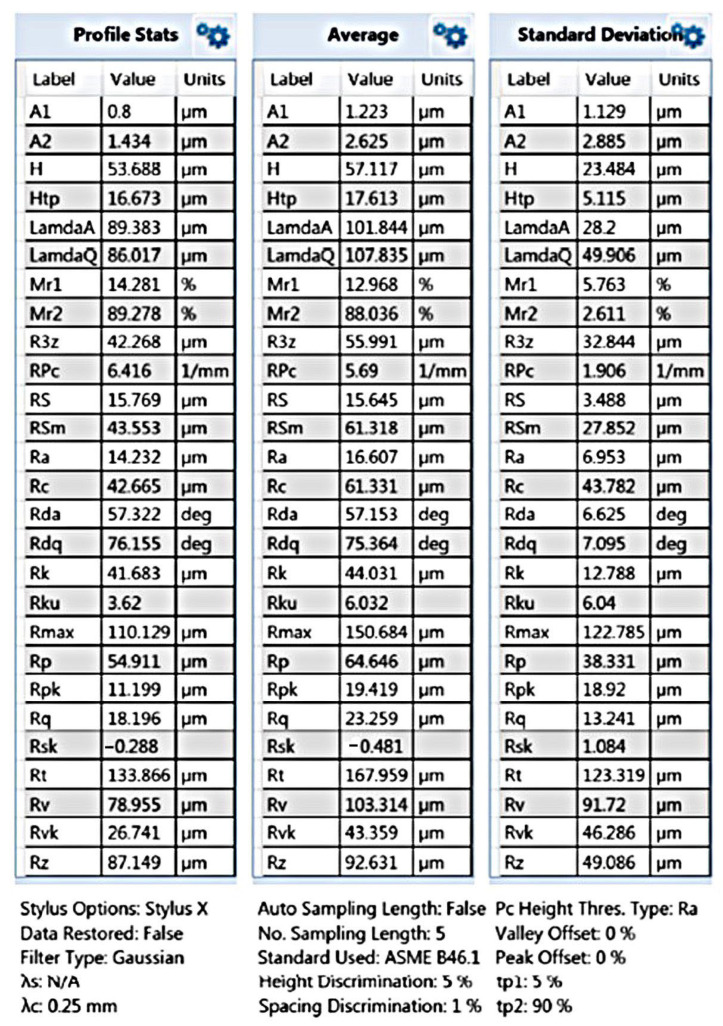
The results of measuring the thread quality before thermal pulse processing.

**Figure 10:**
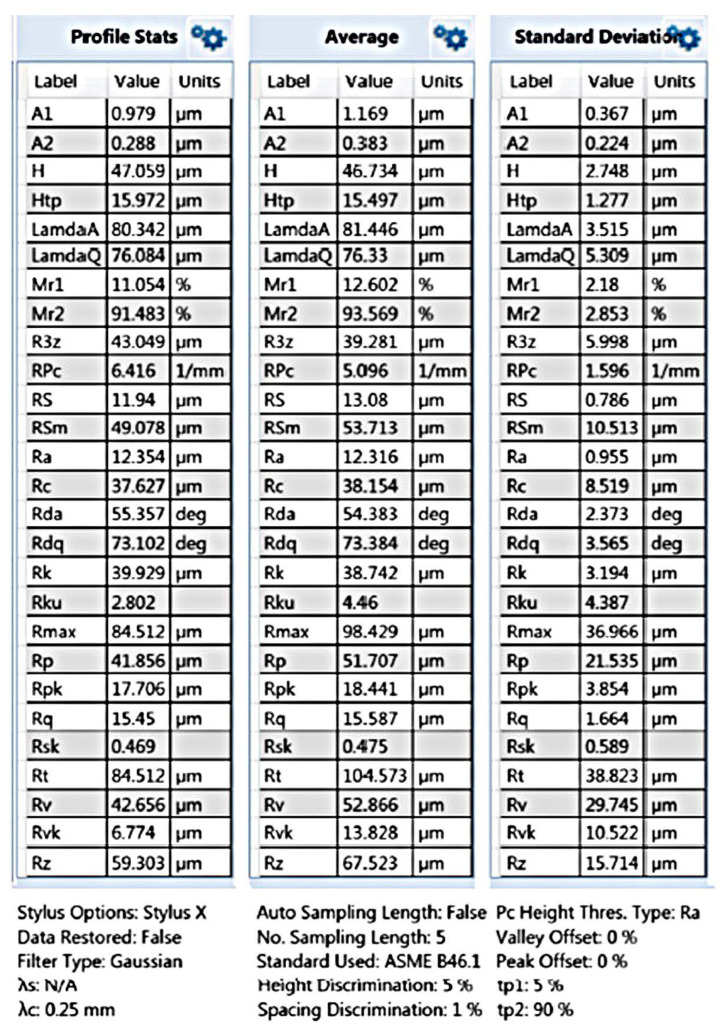
The results of measuring the thread quality after thermal pulse processing.

The results of the analysis of the quality of the thread surface show that the surface roughness decreases (Figure 7, Figure 8, Figure 9 and Figure 10).

The results of measuring the thread quality are due to the smoothing of surface roughness by thermal impulse processing declared by the manufacturer of the installation. As can be seen in Figure 5, Figure 7, Figure 8, Figure 9 and Figure 10, after thermal pulse processing in selected modes, the burrs were removed, the thread geometry was preserved, and the surface roughness did not increase.

Edge rounding does not occur in the smart mode. The use of double processing or processing at increased pressure leads to rounding the edges, but thin-walled and critical elements, such as threads, are damaged. It has been experimentally confirmed that the complete removal of burrs with a root thickness of 0.1 mm from thin-walled parts and the parts with threads (more than 1/4–1/6 of the wall thickness) leads to a violation of the geometry of the parts. For the parts that have thin-walled surfaces, threads, and other small-sized structural elements whose geometry must be preserved, rounding edges and chamfers must be performed at the machining stage.

It has been established that the batch size of loading parts can vary within 25% of the recommended batch size up or down without loss of processing quality. A more significant increase or decrease in the load batch requires the installation of a different processing mode or the use of ballast.

Dependences 1 and 2 are known, which establish the relationship between the parameters of thermal pulse processing and the total area of the treated surface [19].

The volume of the combustible mixture is calculated by the equation:

(1) $$V_{gm}=\frac{V_{wc}\cdot k_{l}\cdot P_{gm}\cdot T_{0}}{T_{gm}\cdot P_{0}}$$

where $V_{wc}$ is the working chamber volume;

$k_{l}$ is the parts load factor;

$P_{gm}$ is the gas mixture pressure; and

$T_{gm}$ is the gas mixture temperature.

At the same time:

(2) $$V_{gm}=\frac{F_{t}\cdot q\cdot \tau }{Q_{gm}}$$

where $Q_{gm}$ is the volumetric heat of gas mixture combustion;

$V_{gm}$ is the gas mixture volume;

$F_{t}$ is the total area of heat-removing surfaces; and

τ is the duration of the heat source.

We may take into account only the parameters that have a significant impact on the process of thermal impulse processing. They are the simplified dependence of the volume of the gas mixture on the volumetric heat of combustion, the area of the processed surface of the parts, and the parameters known for a particular chamber: load factor, temperature, and composition of the gas mixture. Then, we can quite accurately set the basic value of the pressure of the combustible mixture to reduce the number of experiments on the selection of modes [3].

### 3.3. A Rational Option for Filling the Chamber of the Thermal Impulse Installation with Parts to Ensure High-Performance Deburring without Damaging the Surfaces of the Parts

**Figure 11:**
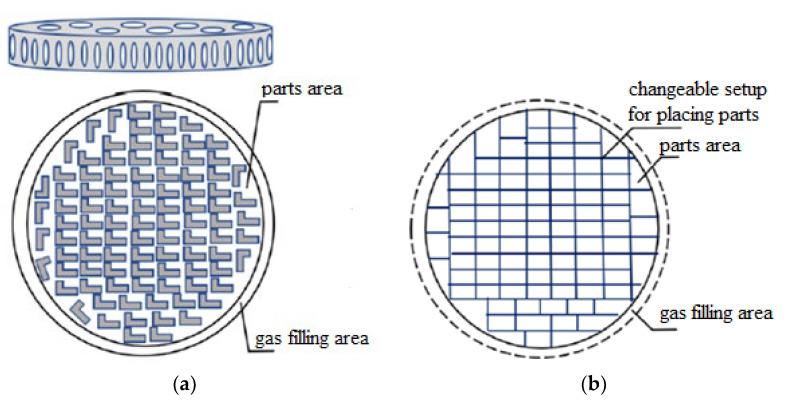
Schematic representation of the rational option for placing parts [3]: (**a**) the base part of the device; (**b**) shift adjustment.

Details of coaxial radio components are small, fragile, and precise. For thermal pulse processing, it is necessary to place parts in such a way as to protect them from damage during an explosion, ensure access of the gas mixture to all surfaces of the parts, and minimize costs for placing parts in the fixture. There are known suspension devices, rocking baskets for small parts, such as “frame”, “herringbone” with spring contact, and tubes with holes for accommodating cylindrical parts, the placement of parts in which is laborious. The most suitable option for placing parts is to use a special setting device for group processing in the form of a mesh basket of small height with a fixed mesh cover for placing parts in one layer using an interchangeable setting, a dividing grid with cells corresponding to the size of the loaded parts. The application of the interchangeable setup will allow using a single base part of the fixture for the entire range of parts (Figure 11).

A rational option for placing parts was chosen based on the values of the criteria described in Table 1.

**Figure 12:**
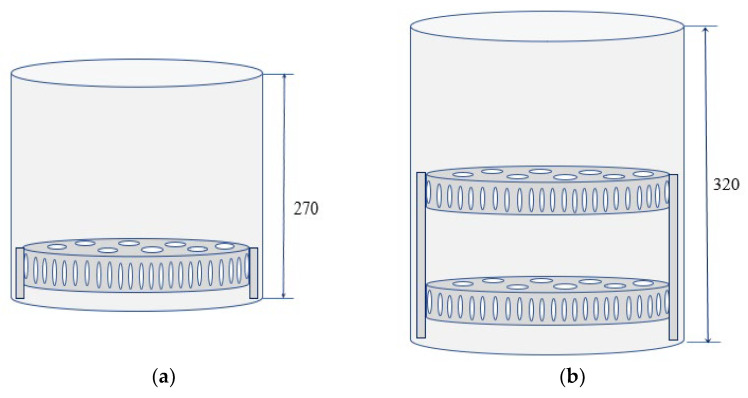
Sketch of the tiered fixture in the working chamber [3]: (**a**) single-tier fixture; (**b**) two-tier fixture.

Due to the provision of increasing pressure in the chamber of the thermal impulse installation, part of the fuel burns out in the detonation mode. The shock wave propagation velocities in the chamber can be up to 2000 m/s. Since during combustion in the detonation mode in a cylindrical chamber, the forces are directed to the cover and bottom of the chamber, the protection of the structure from destruction and the setting of the maximum pressure in the chamber depend on the diameter, and the height of the chamber can be changed. The use of the tiered equipment is proposed, which makes it possible to rationally use the volume of the chamber and multiply the productivity of the installation with an increase in the height of the chamber (Figure 12).

The height of the working chamber of the Pulsar VKF 3.250 thermal pulse installation is 270 mm, the diameter is 250 mm, and the maximum allowable pressure is 1.8 MPa. The required height of the deburring chamber in a two-tier tooling was calculated through the required volume of the gas-air mixture while maintaining a pressure of up to 1.8 MPa. When the chamber height of the thermal impulse installation is 320 mm, the pressure does not exceed the maximum allowable, which is *Pgm* = 1.744 MPa.

## 4. Discussion

On the basis of the conducted studies, a set of technical solutions has been proposed that makes it possible to remove the resulting burrs with high efficiency and high surface quality. For example, for the part “Housing” of a coaxial radio component made of beryllium bronze of the BrB2 brand, the developed solutions allow, in comparison with the technology used in production to remove burrs in a tumbling drum and manual processing, increasing the productivity of processing 2.5 times and eliminating defects due to not removing burrs in hard-to-reach surfaces 1.5 times. For another part, the hermetic radio component bushing made of the 29 NK alloy, the developed solutions make it possible to increase the machining productivity by a factor of 2 and exclude rejects by not removing burrs in hard-to-reach surfaces and gouges 1.7 times.

Based on the results of the study, the following conclusions were formulated:

1. It has been experimentally established that the thickness of the burr root remaining after turning workpieces made of beryllium bronze BrB2 and the 29 NK alloy depends on the parameters of the cutting mode during turning. For beryllium bronze BrB2, the thickness of the burr root is primarily affected by the value of the longitudinal feed during turning, then by the cutting speed and depth of cut. For the 29 NK alloy, the thickness of the burr root depends only on the cutting speed. The difference in dependences is explained by the peculiarities of the physical and mechanical characteristics of these materials, namely, Young’s modulus, strength, and thermal conductivity.
2. The relationship between the completeness of deburring by physical and technical thermal impulse deburring and the dimensions of workpieces, namely, the thickness of their walls, has been established. Complete removal of burrs without the formation of defects on the part is ensured when the thickness of the burr root is within 1/4–1/6 of the minimum thickness of the part.
3. Established rational modes of thermal pulse processing ensure complete removal of burrs while meeting the requirements of the part drawing, the quality of the machined surface and the productivity of processing. The combustible mixture pressure parameter is set taking into account the thermal conductivity of the material of the workpieces, the area of the treated surface, and the characteristics of the installation. If, at the assigned pressure of the combustible mixture, all burrs with a root thickness not exceeding that established for the processed nomenclature at the rate of 1/4–1/6 of the minimum wall thickness are removed from the part occupying 30% of the chamber volume in this installation, then for the parts with the same level of thermal conductivity the pressure of the mixture changes depending on the change in the area of the treated surface.
4. The developed version of filling the chamber of the thermal impulse installation with parts provides high-performance deburring without damaging the surfaces of the parts by limiting movement during processing, providing access to the gas mixture to all surfaces of the parts, providing rational use of the chamber volume, and using interchangeable adjustment for quick placement of parts in the fixture. This option of filling the chamber allows increasing the productivity of processing by a multiple of the number of tiers of the fixture at minimal cost for placing parts.

The main surface treatment of blanks by the thermal pulse method, due to the short-term high efficiency of heat transfer, occurs in the detonation mode for a short period of time of 20–30 milliseconds, which allows minimizing the thermal effect and maintaining the physical and technical characteristics of the material. However, it was noted that the parts after thermal pulse processing have matte surfaces, which indicates the impact of the workpiece material on the surface layer. Details of coaxial radio components at the next stage of processing are subject to electroplating with an alloy having a high electrical conductivity with a thickness of 1–5 microns. Signal transmission in a coaxial line occurs in this highly conductive layer along the inner surface of the outer conductor and along the outer surface of the inner conductor. Therefore, the changes in the electrical conductivity of the surface of the workpieces after thermal pulse processing were not studied. For other parts made of such materials as beryllium bronze and the 29 NK alloy, it will be important to ensure certain characteristics of the material after thermal impulse processing. In practice, often in the design documentation for the product, a requirement for appearance is written, and the part should remain shiny, not matte. For the products made of beryllium bronze, its high electrical conductivity and elasticity are important. Therefore, when expanding the range of the products made of beryllium bronze and the 29 NK alloy, subject to thermal pulse processing, additional studies of changes in the characteristics of the material in the surface layer may be required.

## 5. Conclusions

Thermal pulse processing is a promising method for removing burrs from small-sized parts made of oxidizable materials, such as beryllium bronze and the 29 NK alloy. It has the following advantages: stability, reliability, complete removal of all burrs from all the surfaces of small-sized parts, and low time expenditures. This method provides the low heating of parts and low condensation of metal oxides, the best ratio of the wall thickness of the part to the thickness of the burr.

Conditions have been established that ensure the removal of burrs by the thermal pulse method in compliance with the requirements for deviations in the geometry of parts, for surface roughness and for ensuring the maximum processing performance. These basic conditions include restrictions on the thickness of the root of the burr and a variant of the arrangement of parts in the chamber of the thermal impulse installation, which ensures the prevention of damage to parts during processing and provides access to the combustible mixture to all surfaces of the parts. There is also the value of the pressure of the combustible mixture, depending on the characteristics of the thermal pulse installation, the total area of the treated surface, and the thermal conductivity of the materials of the workpieces.

It has been experimentally established that the use of the thermal pulse method is limited by the thickness of the burr root, while it has been proven that the thickness of the burr root should not exceed 1/4–1/6 of the part wall thickness. The normalization of the burr root thickness is carried out at the stage of blade machining. The thickness of the root of the burrs remaining after turning workpieces made of beryllium bronze BrB2 and the 29 NK alloy depends on the parameters of the cutting mode during turning. The difference in dependences is explained by the peculiarities of the physical and mechanical characteristics of these materials, namely, Young’s modulus, strength, and thermal conductivity.

To expand the range of products made of beryllium bronze and the 29 NK alloy subject to thermal pulse processing, additional studies of changes in the characteristics and properties of the material in the surface layer may be required.

## Figures and Tables

**Table 1:** The choice of a rational option for placing parts.

Type of Fixture for Batch Processing	Surface Area Minimum	Placement Time Is Minimal	Mixture Access to All Surfaces of the Part	Small Parts Range Coverage
Container with lid	no	yes	no	yes
Hanger with snap hook	yes	no	yes	only parts with a hole
Suspension with spring grip	yes	no	no	yes
Mesh tube	no	no	yes	yes
Low height container with lid and parts divider	no	yes	yes	yes

## Data Availability

The data presented in this study are available from the corresponding authors upon reasonable request.
